# Fast FMCW Terahertz Imaging for In-Process Defect Detection in Press Sleeves for the Paper Industry and Image Evaluation with a Machine Learning Approach

**DOI:** 10.3390/s21196569

**Published:** 2021-09-30

**Authors:** Maris Bauer, Raphael Hussung, Carsten Matheis, Hermann Reichert, Peter Weichenberger, Jens Beck, Uwe Matuschczyk, Joachim Jonuscheit, Fabian Friederich

**Affiliations:** 1 Department of Materials Characterization and Testing, Fraunhofer Institute for Industrial Mathematics ITWM, 67663 Kaiserslautern, Germany; raphael.hussung@itwm.fraunhofer.de (R.H.); carsten.matheis@itwm.fraunhofer.de (C.M.); joachim.jonuscheit@itwm.fraunhofer.de (J.J.); fabian.friederich@itwm.fraunhofer.de (F.F.); 2 Group Division Paper, Voith Group, 89522 Heidenheim, Germany; hermann.reichert@voith.de (H.R.); Peter.Weichenberger@voith.com (P.W.); jens.beck@voith.com (J.B.); uwe.matuschczyk@voith.com (U.M.)

**Keywords:** terahertz imaging, nondestructive testing, frequency-modulated continuous wave, press sleeves, paper industry, anomaly detection, machine learning

## Abstract

We present a rotational terahertz imaging system for inline nondestructive testing (NDT) of press sleeves for the paper industry during fabrication. Press sleeves often consist of polyurethane (PU) which is deposited by rotational molding on metal barrels and its outer surface mechanically processed in several milling steps afterwards. Due to a stabilizing polyester fiber mesh inlay, small defects can form on the sleeve’s backside already during the initial molding, however, they cannot be visually inspected until the whole production processes is completed. We have developed a fast-scanning frequenc-modulated continuous wave (FMCW) terahertz imaging system, which can be integrated into the manufacturing process to yield high resolution images of the press sleeves and therefore can help to visualize hidden structural defects at an early stage of fabrication. This can save valuable time and resources during the production process. Our terahertz system can record images at 0.3 and 0.5 THz and we achieve data acquisition rates of at least 20 kHz, exploiting the fast rotational speed of the barrels during production to yield sub-millimeter image resolution. The potential of automated defect recognition by a simple machine learning approach for anomaly detection is also demonstrated and discussed.

## 1. Introduction

Terahertz technologies, and in particular their application in nondestructive testing (NDT) for quality control and/or defect recognition, are on their way into industrial markets and real-world applications in production environments, maintenance tasks, and other areas of quality assessment [1,2,3,4]. The terahertz terminology in these contexts commonly refers to electromagnetic radiation in the region of the electromagnetic spectrum with frequencies between 0.1 and 10 THz corresponding to free-space wavelengths of 3 mm to 30 μm. Terahertz waves offer a number of characteristic properties especially interesting for the NDT investigation of nonconducting materials and industrial components or products made from these materials. First, terahertz radiation can penetrate many common production materials at low absorption rates and good penetration depths, in particular plastics and polymer compounds [4], glass fiber-reinforced (GFR) composite materials [5,6,7,8,9], wood [10,11], paper [12] and cardboard [13], dry and wet paint layers or other coatings [4,14,15,16], and many more. At the same time, the small terahertz wavelengths of few millimeters down to several tens of micrometers constitute an ideal premise for imaging techniques [17,18,19,20,21] with image resolutions on the order of typical, relevant defect sizes in components produced from the above materials. Compared to other established NDT technologies such as ultrasound inspection, X-ray screening and computed tomography, terahertz waves offer the unique combination of low photon energies and low radiation power - making it harmless to biological tissue and safe to be used in industrial contexts - and, due to the electromagnetic nature of the waves, the possibility to be employed contact-free with no need for a coupling medium to penetrate the materials under investigation [3,22]. With suitable materials for quasi-optical components, e.g., the polymers PE and PTFE, the radiation can be easily guided and focused according to the specific context of application with quasi-optical lenses [23], typically produced of low-cost materials such as the polymers PE and PTFE, and diffractive elements [24], or with simple metallic mirrors. In addition, guiding and focusing of the radiation with easy-to-fabricate dielectric waveguide antennas has been demonstrated [25,26]. Combining all the above properties and advantages over other NDT techniques, and the increasing availability of sources, detectors and receivers, terahertz technology has today reached a level of maturity to be implemented in industrial production environments or processes of quality control and to offer a valuable benefit in the optimization of these processes. Among the typical real-world NDT scenarios where in particular terahertz imaging can be (or is already being) employed are packaging control [13], production lines in the polymer an plastics industry for the detection of defects or the inspection of welding processes, manufacturing of GFR composites for lightweight construction [7,8,27] for example in the automotive, aviation and space industry [9,28,29], food inspection [30,31], investigation of thermal and electrical insulation materials [26,32], but also in fields of biomedical applications [33], artwork conservation [34,35,36,37], and many more.

In this article we present a rotational terahertz imaging system, in particular at the example of the in-process inspection and defect detection in the production of large press sleeves (roller covers) for the paper industry. Press sleeves made from polyurethane (PU) are used in the paper industry on large rotating roller presses to extract residual water content from the still wet paper pulp under high pressure. The dimensions of such roller presses commonly reach up to 15 m in length and up to around 1.5 m in diameter. Hence, typical surface areas of 50 square meters and more are covered by the relatively thin press sleeves with several millimeters in thickness. During the manufacturing of the sleeves, the PU material is molded onto large rotating metal barrels and stabilized by an inlaid fine fiber mesh, which is woven onto the barrels prior to sleeve production. When the PU is applied onto the barrels with the fiber mesh around them, small defects in the form of mostly spherical air inclusions with diameters of few millimeters down to less than 0.5 mm can form on the sleeves’ backside in contact with the metal barrels, in particular at the grid crossing points of the fiber mesh. These defects cannot be visually identified until the whole press sleeve is finally removed from the supporting barrels when the whole production process involving many time-consuming steps of surface processing is completed. However, the formation of the defects may influence the structural integrity and lifetime of the press sleeves in the actual paper production. There, downtimes in the production lines due to damaged or worn out press sleeves can easily become very expensive. We developed a terahertz imaging system which can reveal defects on the press sleeves’ backsides at an early stage in the production process when they are still mounted onto the supporting, rotating metal barrels. Hence, faulty sleeves with too many such defects can already be separated out from further surface processing and the terahertz imaging can therefore add great value to the optimization of the sleeve manufacturing saving valuable time and production costs. This application scenario demonstrates in a model way the great potentials and evident benefits the use of terahertz technology can have in industrial contexts where other inspection technologies cannot be easily applied.

Our terahertz system consists of frequency-modulated continuous wave (FMCW) terahertz transceivers based on all-electronic, waveguide-integrated components for the terahertz frequency range. We developed two transceivers with working frequencies around 300 GHz and 500 GHz and with corresponding total sweep bandwidths of 90 and 160 GHz, respectively—we have reported on comparable measurement units in previous publications [28,29]). The FMCW technique enables depth resolved measurements to generate 3D volumetric terahertz images [7,38] of the press sleeves in order to be able to separate and investigate the sleeves’ backsides where the defect formation occurs. We integrate our terahertz transceivers into a linear translation stage which is placed in front of the rotating metal barrels with mounted press sleeves and by linear translation along the barrels rotational axis, we record a spiral imaging path across the press sleeves’ surfaces. In this way, we manage to limit the need for an interaction with the production machines to a minimum level—no direct communication with the rotational mechanics is required—and at the same time we exploit the very high rotation speeds of the sleeve production machine of up to 150 rpm. In order to meet the requirements in terms of image pixel resolution down to around 0.5 mm we tuned our terahertz transceivers to reach data acquisition rates of up to 20 kHz for a single-point measurement. We show in this article that the above concept is suitable for the inline NDT inspection and defect recognition of press sleeves in the industrial production environment.

Since the surface area of the press sleeves is quite large, manual inspection for possible defects over such an area can become very time consuming and the risk of overlooking smaller defects become quite high. On the other hand, the large sleeve area with a relatively limited number of round defects, which show with good contrast in the acquired terahertz images, constitutes a promising situation for the automated image processing and defect detection by machine learning (ML) approaches [39]. Application of ML techniques to terahertz measurements has been reported many times before, however, mostly in terms of direct application of the ML methods to the quite complex terahertz signals (in pulsed time-domain systems or continuous-wave systems [40]) and employing various sophisticated ML concepts such as artificial neural networks (ANNs) [39,41], random forests, support vector machines (SVMs) and many others (see [42] and references therein). There exist only few examples where ML is applied on the acquired terahertz images in an image processing sense, in which a direct evaluation of the image content itself is performed, rather than the measured signals. One reason may well be that large amounts of terahertz image data with reasonable quality - and relevance in terms of realistic, not artificially implemented defects - are often not readily available, as is commonly required for the training of most of the above ML methods. In addition, in many contexts the spectroscopic information contained in the terahertz data may be of great use. However, in defect detection in industrial production environments, often the mere existence of an anomaly showing up in terahertz intensity images may well be sufficient to sort out a product, without any need for further insight knowledge about the precise terahertz signature of the defect. We demonstrate here that the processing of simple terahertz intensity images (measured in reflection or transmission) can already yield enough information for an automated or semi-automated inline quality control. Some examples of ML techniques applied to terahertz images for defect or abnormality detection are References [41,43,44,45]. We note that there exist a number of works on the topic of object recognition and image segmentation in terahertz images, which could possibly be translated to the task of defect detection in production materials and components.

The measurement scenario we present in this work offers two main benefits for the use of automated defect detection in the recorded terahertz images. On one hand, the use of FMCW transceivers allows us to use some a priori knowledge of the investigated samples, namely, that the we can pre-select a specific depth layer where the defects occur (here: the sleeves’ backsides) out of the full volumetric image data, which is acquired. Second, huge, intact sleeve areas are compared to relatively few, small defects and thus, outlier or anomaly detection ML methods should be a natural approach to our specific task of defect recognition. We demonstrate that even with a simple statistical multivariate Gaussian anomaly detection approach [46,47], we can already achieve good detection accuracy on our measured terahertz data sets. Naturally, with increasing operation time of the imaging system in the press sleeve production, large amounts of terahertz image data can be obtained, which could be used for the training of further, more complex ML algorithms. Nevertheless, even with our straight-forward approach we can provide ML-based support to the manual work of quality control personnel.

## 2. Materials and Methods

In this section we describe the details of our terahertz setup for the imaging of paper press sleeves. First, we present the terahertz FMCW transceivers we used for our measurements. We then explain the imaging setup we have realized to obtain 3D volumetric images of large press sleeve areas with very little need to interfere with the actual sleeve production process as desired in the early stage of production where our measurements take place.

### 2.1. Terahertz FMCW Transceivers

For the terahertz imaging of press sleeves we employ two all-electronic, waveguide component-based FMCW terahertz transceivers with operation frequencies around 300 and 500 GHz with sweep bandwidths of 90 and 150 GHz, respectively. We choose these particular transceivers for a good combination of penetration depth in the press sleeves’ PU material and high spatial resolution to identify sub-surface defects on the rear side of the sleeves.

**Figure 1:**
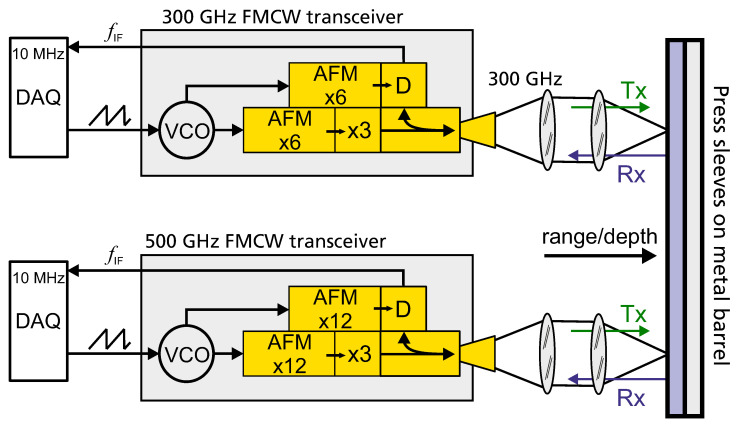
Schematic of the FMCW terahertz transceivers. Linear voltage ramps from a data acquisition unit’s (DAQ) analog output drive the voltage controlled oscillators (VCOs) at frequencies from 12 to 18 GHz for the 300 GHz and 9 to 15 GHz for the 500 GHz system, respectively. The frequencies are then multiplied in waveguide component-based multiplier chains to the desired target frequencies of 230 to 320 GHz and 350 to 510 GHz. We use waveguide horn antennas in combination with quasi-optical lens systems to focus the outgoing radiation (Tx) onto the press sleeves. The reflected radiation (Rx) is collected by the same quasi-optics and guided to Schottky-diode receivers and mixed with the VCOs reference output ramps. The generated difference frequency signals are sampled by 10 MHz ADC input channels of the DAQ.

We employ two slightly different setups in our two measurement units, as shown in the schematics in Figure 1 (a photograph of the two sensor units mounted on top of each other is shown in Figure 3 in Section 2.3). The 300 GHz transceiver uses an active frequency multiplier (AFM) with a multiplication factor of 6 driven by a voltage-controlled oscillator (VCO) to generate linear frequency ramps in the W-Band between 70 and 110 GHz. On the other hand, the 500 GHz transceiver employs AFMs with multiplication factor 12 to generate frequency ramps in the 115 to 175 GHz range. In both transceiver setups, the AFM output is multiplied by another frequency multiplier with multiplication factor 3 yielding sweep frequencies from 230 to 320 GHz and 350 to 510 GHz, respectively. Note that the exact operation conditions and usable bandwidths of the electronics offer some tuning range and depend on the specific components of the multiplier chains and attached antennas.

The terahertz radiation (Tx) is coupled out via directional output couplers with attached horn antennas designed for the respective waveguides of the two frequency bands. We use quasi-optical PTFE-lens systems (50 mm focal length) to focus the terahertz radiation onto the target under test. The reflected terahertz signals (Rx) from the target—in detail: from reflecting interfaces within the terahertz-transparent target—are received by the same quasi-optics and horn antennas of the transceivers and fed to (third) subharmonic Schottky-diode mixers. There, the received signals are mixed with reference frequency ramps generated in a second AFM for heterodyne operation. The resulting intermediate frequency (IF) beat signals $f_{b}$ are sampled in a data acquisition unit (DAQ) at 10 MHz sampling rate—we integrate delay lines in our measurement system to obtain IFs between 1 and 4 MHz to stay below the Nyquist-Shannon frequency of the DAQ. For a single reflecting interface at distance *d* to the transceiver, the sampled beat frequency signal $f_{b}$ directly correlates with the time of flight τ of the received Rx signals compared to the TX reference frequency ramps [38]

(1) $$\tau \frac{B_{\mathrm{sweep}}}{T_{\mathrm{sweep}}}=f_{b},$$

where $B_{\mathrm{sweep}}$ is the bandwidth and $T_{\mathrm{sweep}}$ is the sweeping time of a single linear frequency ramp of the respective transceiver. The distance *d* to the target’s reflecting interface can then simply be deduced from

(2) $$d=\frac{c}{2}\tau =\frac{c}{2}f_{b}\frac{T_{\mathrm{sweep}}}{B_{\mathrm{sweep}}}$$

with *c* the effective speed of light in the material of the object under test and the factor 2 stemming from the measurement in reflection geometry.

For terahertz-transparent target materials, the measurement signal constitutes the sum over all single (and multiple) reflections within the target under test superposed in the receiving mixer. Consider that such multiple reflections can be in particular important, e.g., for signal modeling approaches in high-resolution thickness measurements with terahertz FMCW systems [48]. In terms of signal processing, the real measurement signal sampled in the DAQ is bandpass filtered and converted into an analytical signal, which is subsequently windowed by an appropriate window function and then Fourier-transformed into the frequency domain. A more detailed discussion of the signal processing steps can be found, e.g., in Reference [48].

The theoretical range resolution for the FMCW sensors is again directly related to the sensors’ sweep bandwidth via

(3) $$\Delta r=\frac{c}{2B_{\mathrm{sweep}}}.$$

For our sensor configurations we find maximum range resolutions (in air, n=1) of roughly Δr=1.6mm for the 300 GHz system and Δr=1mm for the 500 GHz system at full sweep bandwidths. We note that enhancement at the same time of range and lateral resolution in FMCW imaging systems by computational image processing has recently been reported [49].

In standard configuration, our terahertz sensors operate over large sweeping bandwidths of 90 and 160 GHz around the 300 and 500 GHz center frequencies, respectively. The duration of one single linear frequency sweep in both cases is 200 μs for 2000 sampling points, mainly defined by the digital-to-analog (DAC) converters of the 10 MHz DAQ unit driving the VCOs. As a result, the typical maximum data acquisition rate for a full frequency ramp without any signal averaging is 5 kHz. However, for the specific application of NDT of large press sleeves for the paper industry, we had to realize significantly higher single-point measurement rates of up to 20 kHz to address the high rotational velocities of the press sleeves and the desired spatial resolutions of the resulting terahertz images (see Section 2.2 for details). We achieve this by cropping the sweep bandwidths $B_{\mathrm{sweep}}$ but keeping the slope $B_{\mathrm{sweep}}/T_{\mathrm{sweep}}$ of the frequency ramps in (1) constant to ensure that the IF frequency remains around 5 MHz satisfying Nyquist’s sampling theorem for the maximum 10 MHz sampling rate of our DAQ. Thus, for an effective 20 kHz data acquisition rate, the remaining sweep bandwidths of the two terahertz sensors amount to 27 GHz for the 300 GHz unit and 45 GHz for the 500 GHz unit. Although the maximum range resolutions after (3) are therefore reduced to 5 mm and 3.3 mm (in air, n=1), respectively, we show in our measurements results below that this is still sufficient for a discrimination of the press sleeves’ front and back sides for defect detection purposes.

Both terahertz transceivers are equipped with quasi-optical focusing lens setups with focal distances of 50 mm. The wavelength limits of the lateral resolution amount to roughly 1 mm and 0.6 mm for the 300 and 500 GHz systems, respectively. We note, however, that in many imaging scenarios, defects with diameters below the theoretical resolution limit of the measurement setup can still be inferred from full 2D cross-sectional image data—especially in cases of spatial oversampling [50]—even though the defects are not fully resolved in the strict sense of the technical term.

Altogether, with the described FMCW measurement approach we obtain single-point terahertz depth-profiles (A-scans) from our terahertz FMCW transceivers at kHz measurement rates. Finally, in order to acquire 3D volumetric terahertz images, the terahertz sensors have to be combined with appropriate scanning mechanics to form 2D depth-profiles along a single line (B-scans) or across a 2D surface (C-scans). In the application scenario presented in this contribution, we employ the FMCW transceivers in a rotational imaging setup, which is described in detail in the following section. For the NDT inspection of press sleeves, this enables us to monitor the hidden backside of the sleeves, where typically the formation of defects occurs during production.

### 2.2. Terahertz Imaging Setup

**Figure 2:**
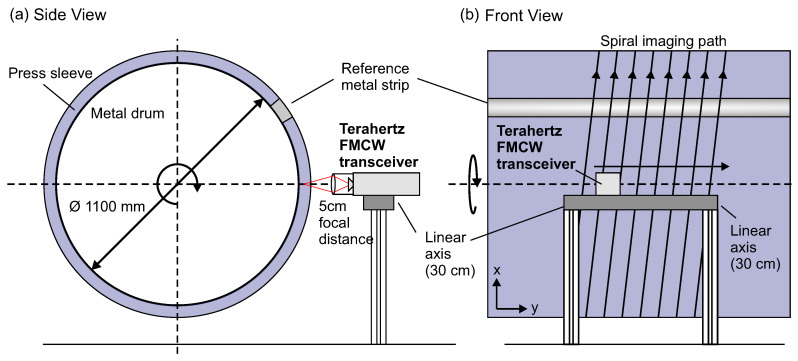
Measurement scheme of the terahertz imaging setup for the investigation of paper press sleeves. (**a**) Side view showing the terahertz FMCW transceiver with 50 mm focusing optics mounted on a linear axis and placed in front of the rotating sleeves at the height of the rotational axis. (**b**) Front view. During measurement, the terahertz transceiver moves along the linear axis and terahertz volumetric data is recorded along the indicated spiral imaging path. An attached metal strip serves as rotation reference with a peak in the terahertz reflection signal on every roundtrip.

Schematic views of our terahertz FMCW imaging setup for NDT inspection of press sleeves for the paper industry are depicted in Figure 2. During production, the PU press sleeves are molded onto large rotating metallic barrels with diameters on a meter scale. In order to obtain volumetric quasi-3D terahertz images of large sleeve areas, the terahertz sensors are mounted on a mechanical translation stage at the height of the barrel’s rotational axis. The sensors are operated in continuous data acquisition mode recording a continuous stream of terahertz FMCW sweeps—i.e., single-point depth profiles (A-scans)—during measurement. As the sensor moves along the linear translation stage while the metal barrel with press sleeve is spinning, a spiral imaging path across the surface is recorded. In the current implementation of the imaging system, no further rotational encoder information is used for automatic synchronization of our terahertz measurements with the metal barrel’s rotation. We therefore attach a small metal strip onto the press sleeves parallel to the translation axis (y axis), which produces a strong spike in terahertz reflection signal on every revolution (x axis). We implemented an edge detection algorithm searching for maximum terahertz signal per revolution to align the acquired stream of terahertz data along the metal strip. In this way, the recorded data is unrolled along the spiral imaging path and 3D volumetric terahertz images of the press sleeves are obtained. Therefore, the measurement setup operates completely independent of the rotating metal barrel with surrounding press sleeves, as long as a constant rotational speed of the barrel is ensured. With this approach we can integrate our measurement system into the given circumstances of the production environment with no further need of higher level communication with the rotational axis of the manufacturing machine.

During production, the press sleeves together with the supporting metal barrels rotate at quite high rotational velocities of up to 150 rpm. We designed our terahertz imaging system in such way that we can exploit these high velocities to obtain terahertz images of the entire sleeve area at reasonable scanning times being defined only by the velocity of the linear translation stage. The fast rotational velocities together with typical sleeve diameters of about 1.1 m result in significant surface velocities of up to 10 m/s of the transceivers scanning along the spiral trajectory across the sleeves’ surface. Therefore, fast data acquisition rates of up to 20 kHz for the full FMCW sweeps are required to realize pixel resolutions of roughly 0.5 mm along the direction of the circumference (y) in the final terahertz images. Note that for a similar resolution along the translational axis, a linear velocity of roughly 1 mm/s is required. For typical sleeve lengths of up to 13 m, the total image acquisition time for an entire sleeve is approximately 4 h. Since we integrate our measurements directly into the manufacturing process, this does not add significantly to the total time of sleeve production and the terahertz imaging can even be combined with mechanical surface processing steps with comparable time consumption.

It should be mentioned that this simple approach comes with some minor downfalls. First, the terahertz images to some extent show some jittering from line to line, because it cannot be guaranteed that the exact moment of passing the metal stripe coincides exactly with the identical position within one frequency sweep performed by the terahertz sensor from one roundtrip to the next. This, however, is a fundamental problem which could not be easily solved by interpolation onto a finer pixel grid. Higher level synchronization with the rotational axis’ motion controller would be required to address this issue. Here, we deliberately did not pursue this approach due to the simplicity of the presented measurement procedure. Second, we rely on the constant velocity of the rotational (and translational) axis to obtain images with constant resolution in y (and x) direction over the whole image. Potential fluctuations in one or both velocities could in principle lead to a distortion in the acquired terahertz images. Nevertheless, we have already successfully demonstrated a comparable imaging approach in a previous work where a 5-axis milling machining was combined with a dual-frequency terahertz sensor to obtain 3D volumetric images of aircraft radomes [28,29] with even more complex conical geometry compared to the cylindrical press sleeves presented here. There, additional position information of the 5-axis machine was used for the alignment of the volumetric terahertz image data after each measurement.

### 2.3. Preliminary Studies on a Laboratory Scale Model

**Figure 3:**
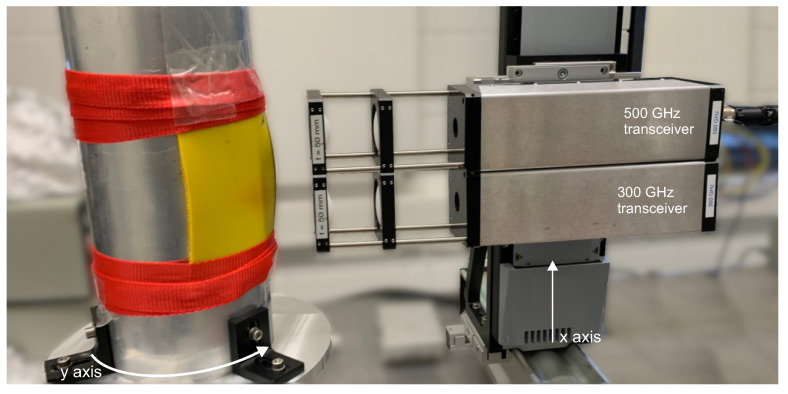
Laboratory-scale realization of the rotational imaging setup. Cut-out pieces of press sleeves are attached to a metal cylinder on a rotational stage. The two terahertz transceivers are mounted on a linear translational axis pointing towards the rotational axis of the metal cylinder. By linear translation along the cylinders axis, terahertz data is recorded on a spiral imaging path.

Figure 3 shows our terahertz sensors set up in a laboratory-scale test setup, which has been designed to mimic the real world situation in the production environment of the real paper press sleeves. We used this setup for preliminary tests on relevant model samples of press sleeves, i.e., cut-out pieces of full-scale press sleeves, which contained real defects of various sizes. For the test setup, we attached our terahertz FMCW transceivers to a vertical linear translation stage, which was placed in front of a rotation table. The terahertz sensors were aligned to point at the vertical rotational axis of the rotation table and could be moved over a linear travel range of 450 mm. We aimed to emulate our final application scenario using a metal cylinder with 30 cm diameter onto which we mounted pieces of press sleeves with real defects from the manufacturing process. With rotational velocities around 60 rpm we reach similar surface velocities as in the full-scale imaging setup described above. We note again that the imaging setup could also be used to record terahertz images of noncylindrical objects as long as a certain degree of rotational symmetry is given. Although the image reconstruction method at this stage relies on a constant surface velocity and an adaptation of the rotational speed during the measurement may not be feasible, it may depend on the requirements of the specific NDT application if a distortion of the terahertz images after data alignment can be accepted at the benefit of this simple and fast imaging concept for rotational symmetric objects.

**Figure 4:**
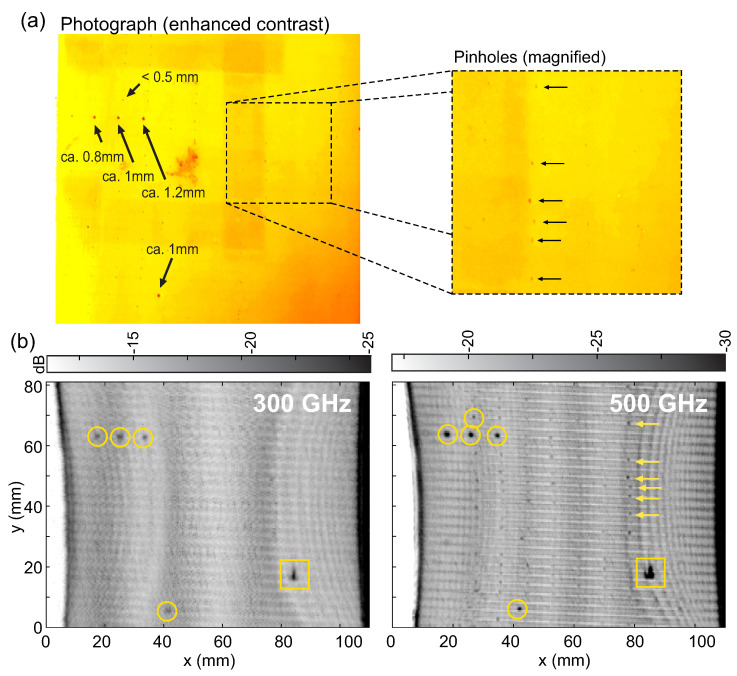
(**a**) Photograph of the backside of a piece of press sleeve with several hole defects of different diameters. The magnified section of the image shows a number of small pinhole defects with <0.5 mm diameter, indicated by the arrows. (**b**) Terahertz images of the investigated sample showing the hidden backside of the sleeve facing the metal cylinder when mounted as in Figure 3. The images were recorded at a data acquisition rate of 20 kHz with the two terahertz transceivers at 300 GHz and 500 GHz center frequency. Defects down to 0.8 mm (circles) as well as an unexpected larger defect inside the sleeve (rectangle) can be detected with both systems. The imaging system working around 500 GHz can even reveal a large number of the pinhole defects, the ones marked with yellow arrows corresponding to the pinholes in (**a**).

The measurement process with our laboratory setup works similar to the method described above in Section 2.2 for the final application scenario, except that for testing purposes, only a segment of a press sleeve was used. Note in Figure 3 that the press sleeve segment only partially covers the metal cylinder and the transition from the press sleeve to the metal cylinder itself (instead of an additional metal strip as in the final setup) serves as the reference metal edge for the terahertz data alignment. Figure 4a shows a piece of paper press sleeve we used as a model sample for the preliminary tests. The sample shows a number of defects, namely, clearly visible larger defects of sizes from roughly 1.2 mm to 0.8 mm (measured with a mechanical caliper), and a number of smaller pinhole defects with less than 0.5 mm in diameter. The defects tend to form at the cross-section points of the sleeves’ fiber mesh inlays and thus are arranged rather regularly across the sample area. The magnified photograph shows the regular arrangement of some pinhole defects. We mounted the piece of press sleeve on the metal barrel as shown in Figure 3 and performed terahertz measurements with both FMCW transceiver units at 300 and 500 GHz operation frequencies. The measurements were recorded at a data acquisition rate of 20 kHz at a rotational speed of 60 rpm and a translational velocity of 0.5 mm/s of the linear axis. With these parameters, we achieve a surface pixel size of approximately 0.5 mm along the circumference (x axis) and 0.5 mm along the linear axis (y axis). Recall that with a realistic diameter of the metal barrel of up to 1.5 meters and rotational velocities of up to 150 rpm, the resolution of the final measurement setup calculated to approximately 0.5 mm along the circumference and 0.2 mm along the linear axis.

Terahertz images of the test sample are shown in Figure 4b. The images represent cross-sections (C-scans) of the press sleeve samples a the depth close to its backside in contact with the metal cylinder at around 8 mm below the outer surface. In both measurements, the defects down to a size of roughly 0.8 mm can be clearly recognized (marked by the yellow circles). However, the measurement at 500 GHz also reveals most of the smaller pinhole defects distributed along the regular grid crossing points of the fiber mesh inlay (marked by yellow arrows in the figure and corresponding to the pinholes in Figure 4a). Thus, when penetration depth and/or dynamic range of the 500 GHz transceiver unit are sufficient (which is in particular the case for the PU press sleeves), our measurement system can detect small defects even slightly below the corresponding free-space wavelength of 0.6 mm. We observe that the terahertz images are overlaid by a number of interference fringes, which were caused by a deformation of the press sleeve being stretched onto the metal cylinder by the help of two tension belts - the cut-out sample with original sleeve diameter of 1.1 m could not be mounted perfectly flat onto the smaller diameter of the lab-scale metal barrel. In the final measurement scenario, such interference patterns are not present in the terahertz images (see Section 3.1). We also note that a larger material defect is found inside the sleeve material (marked by the yellow rectangles) in the lower right corner of the test sample which was not expected from visual inspection. This underlines once more the great value of terahertz NDT imaging in general for the detection of hidden defects inside terahertz-transparent materials.

We finally note that the costs of waveguide components for all-electronic terahertz FMCW transceivers usually grow significantly with desired output frequency and a trade-off between required detectable feature sizes depending on the particular application and hardware costs of the measurement system should be considered. For our scenario of defect detection in paper press sleeves, we find the 500 GHz FMCW transceiver to best meet the requirements of the specific application. Nevertheless, the above results also prove the applicability of the 300 GHz measurement unit when slightly less spatial resolution of the imaging system may be sufficient.

## 3. Results and Discussion

In this section we present terahertz imaging results of on-site measurements of full-scale press sleeves for the paper industry in a typical production environment. The resulting image data was first investigated manually to assess the feasibility of the measurement approach. We find that the imaging system with the 500 GHz terahertz transceiver yields promising results to be used for an inline NDT detection of typical defects in press sleeve production. We also applied rudimentary machine learning to the acquired terahertz images to demonstrate the future potential of automated defect recognition, in particular of interest for the scenario of the inspection of large press sleeve surface areas, where a manual quality control can be quite complex and time consuming.

### 3.1. Measurement of Press Sleeves in Real-World Scenario

**Figure 5:**
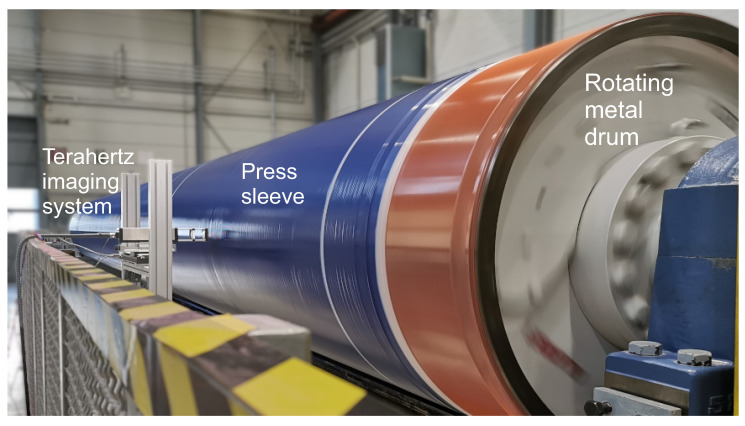
The terahertz imaging system setup in a realistic production environment of press sleeves for the paper industry. The sleeves are investigated within the usual production process to detect possible invisible defects at an early stage of the production line. An area of around 1 square meter of sleeve surface was recorded in this study, limited only by the total travel range of the linear translational axis. Due to the high rotational velocities of up to 150 rpm and large sleeve diameters up to 1.3 m, the terahertz FMCW transceivers are operated at very high data acquisition rates of 20 kHz to yield desired image resolutions of around 0.5 mm.

We installed our terahertz imaging system in a real production environment of press sleeves for the paper industry at Voith Group. Figure 5 shows a photograph of the system during the measurement of a press sleeve on the rotating metal barrel. In this particular setup, the terahertz transceiver was mounted on a linear stage with a travel range of 30 cm to scan over a total area of approximately one square meter of the sleeves surface. For the first imaging run, the rotational speed of the supporting metal barrel was set to 60 rpm and the linear axis’ velocity was 0.5 mm/s, yielding image pixel resolutions of 0.2 mm along the circumference (x axis) and 0.5 mm along the linear axis (y axis). With a sleeve diameter of 1.1 m the total imaging area was approximately 1 square meter. The time of image acquisition with the above settings amounted to roughly 10 minutes when scanning over the full 30 cm travel range of the linear stage.

**Figure 6:**
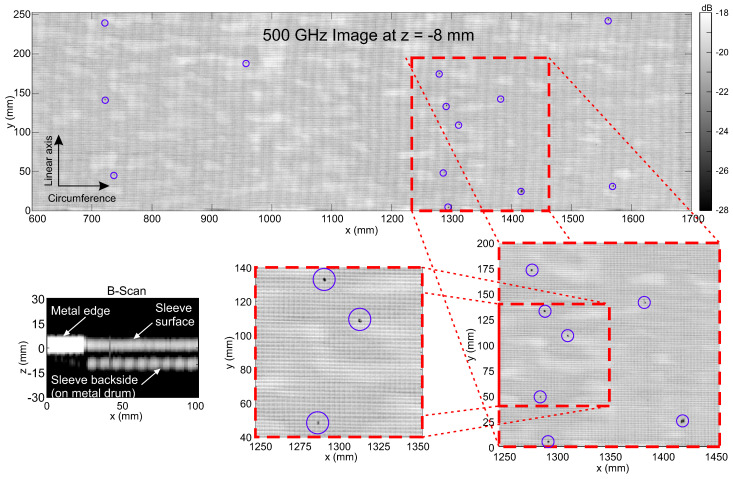
Terahertz image (C-scan) of a larger segment (1100 × 250 ${\mathrm{mm}}^{2}$) of a press sleeve measured at the production site as in Figure 5 with the 500 GHz transceiver at 20 kHz measurement rate. The image shows a depth layer close to the sleeves backside in contact with the metal barrel (see B-scan in lower left corner) at 8 mm below the surface. The magnified images indicate that the size of most of the defects is comparable to the grid generated by the sleeve’s fiber mesh inlay roughly amounting to approximately 1 mm in diameter or smaller.

Figure 6 presents terahertz images of a press sleeve acquired in a real production environment of the sleeves as discussed above and as shown in Figure 5. The wide image at the top shows a segment of 25 × 110 ${\mathrm{cm}}^{2}$ out of a total scan area of 1 square meter of the press sleeve—note that it is not possible to display terahertz images of the entire measured surface with reasonable detail on a typical high-resolution computer screen (or paper printout), since the actual size of relevant defects would be well below the size of a single pixel. For a visual inspection of the terahertz images, a possible scenario could be to show segments of the full sleeve area in a continuous scrolling video mode (for an example, please see Supplementary Materials).

The image in Figure 6 represents a cross-sectional layer (C-scan) of the sleeve area at a depth of 8 mm below the sleeve’s surface. The terahertz data was acquired with the 500 GHz transceiver at 20 kHz measurement rate. Recall from Section 2.1 that in this configuration, the depth resolution amounts to approximately 3.3 mm for a single layer. A cutout of a single line scan (B-scan, different press sleeve) is shown in the bottom left image in the same figure. The bright signal on the left of the B-scan represents the reference metal edge on the sleeve’s surface (z = 0 mm) used for alignment of the measured terahertz data. Next to the reference edge, the terahertz data of the press sleeve is seen, with the top layer representing the sleeve’s surface and the layer below representing the signal at the sleeve’s backside at the interface with the supporting metal barrel.

A number of defects can be identified distributed over the terahertz image of the selected sleeve segment as marked with blue circles in the figure. For better visibility, two segments of 200 × 200 ${\mathrm{mm}}^{2}$ and 100 × 100 ${\mathrm{mm}}^{2}$, respectively, are shown on a magnified scale below the full image. It can be recognized that the size of most of the defects is roughly comparable to the size of the rectangular background pattern generated by the sleeve’s fiber mesh inlay. We therefore assess that pinhole defects down to at least 1 mm in diameter can be reliably detected by our terahertz imaging system and even smaller defects may be identified. It should be noted that detected defects need not necessarily be located at the sleeve’s rear side when they appear in the cross-sectional C-scan images at the respective depth layer but may represent shadows of invisible defects within the PU material (compare Section 2.1).

**Figure 7:**
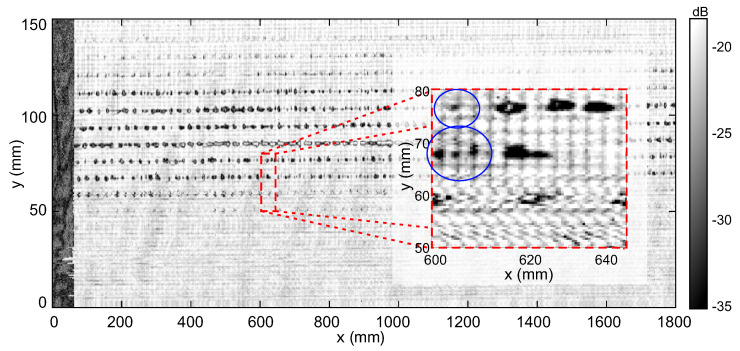
Terahertz image of a press sleeve measured under real operation conditions (150 rpm rotational speed, sleeve diameter 1.3 m) with the 500 GHz FMCW transceiver. The production conditions were deliberately altered during manufacturing to produce this high density of defects in the investigated area. The image shows again only a segment of 1800 × 150 ${\mathrm{mm}}^{2}$ out of the total scan area of 1.2 ${\mathrm{mm}}^{2}$ for better visibility. Clearly, defects of various sizes are revealed to dimensions down to less than the grid spacing of approximately 1 mm of the visible fiber mesh pattern (see blue circles in the magnified inset). The black area on the left of the image represents the shadow of the attached metal strip on the sleeve’s surface for the alignment of the continuously recorded terahertz data.

We present another imaging result of another press sleeve in order to demonstrate the feasibility of our terahertz NDT approach under real operation conditions of the production environment. Before, during the molding process of the sleeve material, the velocity of the production machines was deliberately perturbed to implement typical production defects at a defined area on the sleeve’s backside for our measurement campaign. The press sleeve had a diameter of 1.3 m and we performed our measurements at a realistic rotational speed of 150 rpm of the metal barrel, where the full 20 kHz data acquisition rate of the terahertz transceivers is required to achieve the relevant lateral image resolution of approximately 0.5 mm per pixel in both directions. The image in Figure 7 shows again a cutout segment of the total 0.3 × 4 $m^{2}$ measured sleeve surface area, where the area with the forced production defects between 50 and 120 mm in y-direction is clearly revealed by the terahertz imaging system. The magnified inset shows again that defects down to the (sub-)millimeter scale and the sleeve’s fiber mesh inlay are well resolved even under these real-production conditions. Note that the metal edge reference appears as dark area on the left because the depicted image shows the sleeve’s backside while the terahertz radiation was blocked by the metal edge attached to the sleeve’s surface.

### 3.2. Automatic Detection of Defects

In the above results, we have demonstrated that our terahertz FMCW imaging system can well visualize typical pinhole defects of millimeter sizes during the production of press sleeves for the paper industry. However, it can be a quite complex task to manually investigate typical full sleeve areas of 50 $m^{2}$ and more. Therefore, an automated defect recognition is desired, which can help NDT personnel with a pre-selection of interesting sleeve segments. If possible, a fully automated image processing may even render redundant the need for any manual assessment of the acquired terahertz data, once a reliable detection model has been trained. Here, we briefly show promising results of the application of a simple machine learning (ML) approach for outlier or anomaly detection in the terahertz data acquired during our measurement campaign.

For the automated defect detection, we implemented a statistical anomaly detection model based on a multivariate Gaussian distribution. Before the actual training of the model, we make use of the volumetric nature of our recorded FMCW terahertz data and pre-select the depth layer as displayed before in Figure 6. We split the full recorded terahertz image from the measurement described in Section 3.1 into rectangular image segments of 100 × 50 pixels to generate a total of 1740 samples. Since we apply supervised, statistical anomaly detection for the training of the algorithm, we manually label the image segments where a defect could be visually identified as positive (1) and the remaining samples without defects as negative (0). In the data of the press sleeve shown in Figure 6 we find 25 image segments with visible defects. Due to the extremely low positives-to-negatives ratio in the data set, a simple anomaly detection approach based on a multivariate Gaussian distribution should already yield reasonable precision and recall for automated defect detection. For such an approach, it is beneficial when the training data contains as few outliers as possible. We therefore select a total of 60% of the data samples from areas of the sleeve containing only a total of 3 positive labels (0.3% ratio) for the training of our model and the remaining 40% of the samples containing a total of 23 positive labels (3.3% ratio) as a cross-validation set.

**Figure 8:**
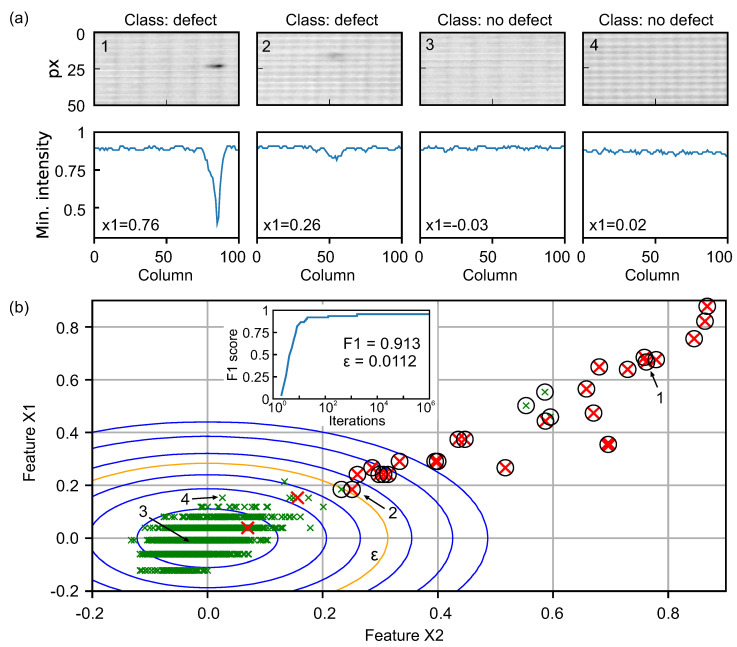
(**a**) Some examples for image segments manually labeled as positive (class: defect) or negative (class: no defect)). The plots below the images show the minimum intensity values per column, corresponding to significant values in feature X1 (standard deviation of minimum value), where true defects are present. (**b**) Two-dimensional feature space of the data set. Green (red) crosses represent samples manually labeled as positive (negative). Contour lines of the multivariate Gaussian probability distribution after training plotted at integer powers of 10 (outwards from +3 to −3) times the decision boundary ε plotted as yellow line. The black circles mark the samples classified as outliers (defects) by the anomaly detection algorithm. The inset shows the good F1 score of 0.913 reached at around 3000 iterations of optimization.

We then define a two-dimensional feature-space for the terahertz data based on the intensity values of each image segment as obtained from the data of the terahertz transceivers. Recall that by using the image from Figure 5 we have already pre-selected a slice from the full volumetric data set where possible defects usually appear on a mostly homogeneous background overlaid with the weak regular grid pattern of the fiber mesh inside the sleeve. Our first data feature X1 represents the total dynamic range of each sample, which should be a good indicator for areas with general inhomogeneities in the sleeve material. However, we define a second feature X2 as the standard deviation of the minimum signal value per column in order to discriminate between an overall distribution of higher dynamic range (e.g., due to the regular grid pattern) and highly localized low intensity values. Hence, we calculate our models input features as

(4) $$\begin{array}{cc} X1 & =\log(\max(X)-\min(X)),\\ X2 & =\log(\mathrm{std}(\min(X,dim=1)), \end{array}$$

where *X* is the set of training or cross-validation samples, respectively, and scale our features by mean normalization. To illustrate our choice of features, Figure 8a gives some examples of image segments with defects (and non-defects) detected by our algorithm in the top row. In the bottom row, the according minimum intensity per column of the above images is plotted. It can be recognized that typical pinhole defects as in example 1 produce a narrow spike in minimum intensity which has a high standard deviation and thus large X2-value. On the other hand, e.g., image 4 may show slightly increased dynamic range due to the regular mesh pattern but has small standard deviation of the minimum value.

Figure 8b visualizes the feature space of our data set and results of the outlier classification with the above parameters. Green crosses represent the samples manually labeled as non-defect and red crosses represent samples manually labeled as defect. We train a multivariate Gaussian distribution with the selected training set with little positive (defect) samples to obtain an optimal Gaussian fit to the large, defect-free areas of the press sleeve as basis for the anomaly detection model. The distribution is naturally centered around zero due to the feature scaling (numerical expectation values are μ = [−2.033 × 10${}^{-16}$, −1.08 × 10${}^{-16}$]) and has variances $\sigma^{2}=\left[0.005,0.006\right]$ in X1 and X2, respectively. In order to define the decision boundary for labelling samples as outliers, we maximize in an iterative process the F1 score of our model applied to the cross-validation data set with high positives-to-negatives ratio. As usual, the F1 score is defined as

(5) $$F1=\frac{\mathrm{tp}}{\mathrm{tp}+0.5\left(\mathrm{fp}+\mathrm{fn}\right)},$$

with tp, fp, and fn the total amount of true-positives, false-positives, and false-negatives, respectively. The F1 score saturates at a value of 0.913 after around 3000 iterations (see inset in Figure 8b) and we find an optimal probability decision boundary of ε=0.0112 for our outlier detection, illustrated by the orange contour line in the figure.

**Figure 9:**
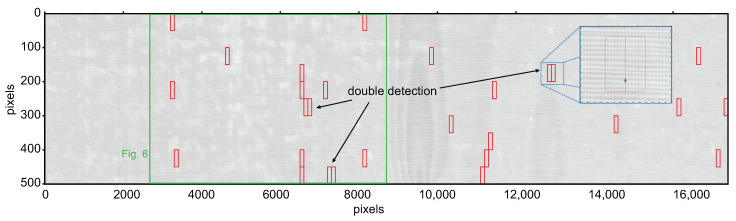
Illustration of the outcome of the automated defect recognition on the whole measured press sleeve area with the previously trained ML anomaly detection model. The red rectangles mark the image segments labeled as outliers (defects) by the algorithm. Double detection occurs when the defects extend from one image segment to an adjacent one (see magnification). The large green rectangle marks the section of the sleeve, which was displayed before in Figure 6. In total, 24 out of 26 manually labeled defects are correctly recognized as outliers, yielding a detection accuracy of 92%.

Finally, when we apply the anomaly detection to the whole terahertz image of the press sleeve, we detect 28 image segments with defects compared to manually labeled 26 positive samples. The detected outliers are marked by black circles in Figure 8b and the respective images segments are marked by red borders in the terahertz image in Figure 9. Note that some of the defects lie on the borders of the 100 × 50 pixels image segments and are detected as two different defects by the algorithm (for example, see magnified inset with blue borders). In a real application scenario, such a doubling should not be of major concern, as long as the defects are detected at all. In total, we find that our automated defect detection misses only 2 out of the 26 manually labeled image segments containing real defects yielding a defect detection accuracy of 92% in the given press sleeve measurement. We note that after omitting the double detections, no further false positive detections occur in this given data set. Thus, with even this simple ML approach we achieve good reliability to support manual inspection of large area press sleeves in the production process by automated defect recognition. Nevertheless, numerous methods exist to further improve the reliability of ML-based anomaly detection, e.g., one-class SVMs [51], convolutional neural networks (CNNs) [52], histogram methods, auto-encoders etc., in multiple variants for semi- or unsupervised learning. An extensive review and further reading can be found, e.g., in Refs. [46,47] and references therein.

## 4. Conclusions

We presented in this work the successful implementation of a terahertz imaging system for the NDT inspection of press sleeves for the paper industry. Defects in the sleeves’ PU material may form at an early stage of manufacturing on the sleeves’ inaccessible backside, which stays in contact with a supporting metal barrel during the whole production process. This makes a visible inspection impossible before the production of a sleeve is finished and it is removed from the barrel. In order to overcome this issue and to prevent unnecessary productions costs for eventually faulty press sleeves, our imaging system can be directly integrated into the production process to identify possible defects at a very early stage of production. We designed our inspection system based on all-electronic FMCW terahertz transceivers working at 300 GHz or 500 GHz center frequencies, respectively, optimized for high data acquisition rates up to 20 kHz. Therefore, we can exploit the fast rotational speeds of the sleeves mounted on the rotating metal barrels during manufacturing, to yield high spatial image resolutions of approximately 0.5 mm, and at the same time achieving reasonable image recording times for the large surface areas of typical press sleeves (typically tens of square meters) with large diameters up to 1.3 m and lengths up to 15 m. Since the terahertz measurement can be integrated at almost any point in the production line, the measurement time itself does not add significantly to the total time of sleeve production. Our study showed that an inspection for defects should - besides aspects of optimization of the production routines and time/cost savings - be performed as early as possible after the molding of the sleeves before any further surface processing (e.g., milling of drainage grooves) has occurred.

We demonstrated the feasibility of the general measurement concept in laboratory-scale studies, where we were able to detect typical defects of diameters down to approximately 0.8 mm and under good conditions of even smaller pinhole defects which can form at the crossing points of a fiber mesh inlay inside the PU sleeves. In order to achieve the high data acquisition rates for the required high surface velocities of up to 15 m/s of the rotating sleeves, we reduced the standard bandwidth of our FMCW transceivers to a level, where we could still separate front and backside of the sleeves via the FMCW principle but could also improve the measurement rate from our usual 5 kHz up to 20 kHz. We then performed a number of measurements in a real production environment and showed the good performance of our NDT imaging system also under these conditions. At the current stage, an attached metal strip is employed to align the continuously recorded terahertz data in to 3D volumetric terahertz images. While this concept requires no access to the rotational mechanics in the sleeve production, using available information of an additional rotation encoder could in the future improve this process to be even less prone to adjustment errors of a reference edge. We demonstrated measurement results obtained with limited translational axes of a maximum of 30 cm length to image a sleeve surface area of 1.2 $m^{2}$. In our ongoing work, we are integrating a larger linear stage to be able to image much larger lengths of the press sleeves in a single measurement, where also a continuous display of the already measured sleeve area in the form of, e.g., waterfall diagrams, can be implemented for continuous process monitoring. Currently, linearly moving milling tools are already being used for the mechanical processing of the sleeves’ surface along a spiral path, very similar to the imaging path of our terahertz sensor. In the future, our relatively compact terahertz transceivers could possibly be mounted directly to the same sliding platforms as the milling tools, to easily gain access to the full length of the press sleeves.

In a first and rudimentary implementation of a ML algorithm for anomaly detection, we demonstrate that the acquired terahertz images are ideally suited for an automated image processing and defect recognition task. Large homogeneous sleeve areas containing only relatively few and small defects can make a manual inspection of the acquired terahertz images quite challenging. On the other hand, this low defect-to-non-defect-ratio constitutes a promising starting point for typical approaches of anomaly detection. We showed in this work that with a two-dimensional multivariate Gaussian fit, we could train an anomaly detection algorithm to yield very high detection accuracy on the measured press sleeves. We also applied the trained model to other areas of the press sleeve with even fewer defects (data not shown) and achieved comparably good defect detection rates. This underlines that with relatively simple ML approaches, the NDT of large area press sleeves can to a large extent be automated, in order to support the work of quality control personnel. Again, an early stopping of the production process, when relevant hidden defects are detected, can greatly enhance the production efficiency in terms of cost and time consumption. In our current implementation, we do not correct for double detection of defects, multiple defects within one image segment, and other artifacts mainly introduced by the current choice of image segment size and definition of the classes “defect” and “no defect”. Optimization of the model’s hyperparameters and error handling of the above cases could further improve the detection accuracy of the proposed ML approach. With growing amounts of terahertz data of press sleeves, more complex ML algorithms could be investigated to even allow for an unsupervised learning of various types of defects for a more sophisticated assessment of relevant irregularities in press sleeve production.

## Data Availability

The data presented in this study are contained within the article.

## Supplementary Materials

The following are available online at https://www.mdpi.com/article/10.3390/s21196569/s1, Video S1: Continuous scrolling video mode.
